# Correlation between Micronutrient plasma concentration and disease severity in COVID-19 patients

**DOI:** 10.1080/20905068.2020.1870788

**Published:** 2021-01-18

**Authors:** Abdullah Alkattan, Khaled Alabdulkareem, Amr Kamel, Heba Abdelseed, Yousef Almutairi, Eman Alsalameen

**Affiliations:** a Department of Biomedical Sciences, College of Veterinary Medicine, King Faisal University, Al-Hofuf, Al-Ahsa, Saudi Arabia; b Department of Research and Development, General Directorate of Medical Consultations, Ministry of Health, Riyadh, Saudi Arabia; c Department of Family Medicine, College of Medicine, Al-Imam Mohammad Bin Saud Islamic University, Riyadh, Saudi Arabia; d Department of Research, Assistant Deputy Ministry for Primary Healthcare, Ministry of Health, Riyadh, Saudi Arabia; e Department of Ghubairah Mobile Team, Tuberculosis Control Program, First Health Cluster, Ministry of Health, Riyadh, Saudi Arabia; f Department of Pharmacy, King Khaled University Hospital, Medical City King Saud University, Riyadh, Saudi Arabia

**Keywords:** COVID-19, sars-CoV-2, micronutrients, immune system, oxidative stress

## Abstract

**Objectives:**

Coronavirus Disease 2019 (COVID-19) is caused by a new strain of betacoronavirus called SARS-CoV-2, which leads to mild to severe symptoms. Micronutrients in blood serum, namely, zinc, iron, copper, and selenium, play essential roles in the human body’s various organs. This study investigates the association between micronutrient levels and the severity of symptoms in SARS-CoV-2 infected patients.

**Methods:**

A cross-section study was conducted during June–August 2020 in Riyadh city among 80 patients with confirmed SARS-CoV-2 infection. Within 24 hours of hospital admission, patients have been divided into non-severe and severe cases, and blood samples were drawn from each patient to measure the serum levels of copper, iron “in the form of ferritin,” selenium, and zinc.

**Results:**

In both study groups, the mean copper and selenium serum levels were within the normal range, while the mean zinc and iron serum levels were elevated. A statistically significant difference was recorded between non-severe and severe cases regarding serum levels of iron and selenium (331.24 vs. 1174.95 ng/ml and 134 vs. 162 mcg/L, respectively, P < 0.0001). On the other hand, no significant difference was detected between both studied groups regarding serum level of zinc and copper (124.57 vs. 116.37 mcq/L and 18.35 vs. 18.2 mcmol/L, respectively, P > 0.05).

**Conclusions:**

There was a significant elevation of selenium and iron serum levels among severe cases compared to non-severe cases of COVID-19. High levels of iron and selenium could be correlated with the disease severity during infection with SARS-CoV-2.

## 1. Introduction

Coronavirus Disease 2019 (COVID-19) is caused by a new strain of betacoronavirus called SARS-CoV-2 (Severe Acute Respiratory Syndrome-Coronavirus-2). The SARS-CoV-2 infection first appeared in Wuhan, a large Chinese city, in late December 2019 and spread globally, leading to the current pandemic [1].

Pathophysiology of COVID-19 involves inflammation of the upper and lower respiratory tract, causing mild to severe symptoms that include fever, dry cough, and shortness of breath. The severity of symptoms depends on the immunity of the infected person. Patients with chronic diseases or those who use immunosuppressant agents are at a higher risk of having severe symptoms of COVID-19 [2].

Micronutrients in blood plasma, namely, zinc, iron, copper, and selenium, play essential roles in the human body’s various organs. Micronutrient blood plasma levels must be in the normal range to guarantee better immune response against different pathogens, including SARS-CoV-2 [3].

The CD8 T-cells of the human’s immune system are the common defensive mechanism against various microorganisms, especially viruses. The activation process of CD8 T-cells could be down-regulated in patients with uncontrolled diabetes, cancer, hypertension, asthma, chronic kidney disease (CKD), autoimmune disorder, cardiovascular disorder, and the use of immunosuppressant agents [4].

Microelements including copper (Cu), iron (Fe), selenium (Se), and zinc (Zn) are substantial elements found in the blood plasma, which have various roles in human tissues and are essential in regulating immune system function. Copper is responsible for interleukin-2 (IL-2) production, which is subsequently accountable for CD8 T-cells activation. Copper deficiency is, therefore, most likely to decrease immunity against viruses [5]. Iron is involved in producing molecules called iron-sulfur clusters, which are cofactors for many vital enzymes involved in DNA repair, citric acid cycle, and lipoyl synthesis. Iron is therefore needed to ensure the regular division of lymphocyte cells during viral infections [6]. Selenium is a cofactor for many enzymes involved in anti-oxidant, anti-inflammatory, and redox mechanisms. Besides, normal blood plasma levels of selenium enhance cellular immune functions and increase the synthesis of cytokines and lymphocytes [7]. Zinc is needed to regulate adaptive immune cells’ functions. Higher intracellular zinc levels increase intracellular pH, affecting RNA polymerase and decrease the replication mechanism of RNA viruses [8].

During some severe bacterial and viral infections, the serum level of zinc was increased because of oxidative stress, increased ferritin level, and cellular damage secondary to these infections, which resulted in increased zinc released from damaged cells into blood plasma [9]. The excess zinc concentration in plasma could lead to the inhibition of pyruvate kinase and mitochondrial complex I, which results in increasing the level of reactive oxygen species (ROS) and decreasing the level of adenosine triphosphate (ATP) [10]. Excess zinc concentration’s net effects include respiratory tract disorders, gastrointestinal tract disorders, focal neurologic defects, copper deficiency, and impairment of lymphocytes’ functions [11].On the other hand, selenium level during bacterial and viral infection was not studied, but its deficiency or excess in blood serum could affect the patient’s health condition. Normal selenium level in blood serum results in reducing cancer risk, oxidative stress, and diabetes risk and improving immune function and male fertility [12]. Elevated selenium serum level because of increased selenium intake or increased inhalation of selenium produced by some industries could lead to selenosis; which characterized by nausea, diarrhea, hypotension, dyspnea, hypersalivation, and garlic breath odor due to the excessive formation of ROSs and endoplasmic reticulum (ER) stress that leads to oxidative stress and cell death [13].

This study aims to determine the association between zinc, iron, copper, and selenium blood plasma levels during viral infection and the severity of symptoms in SARS-CoV-2 infected patients.

## 2. Research design and methods

### 2.1. Study design and study participants

A cross-section study was conducted during June–August 2020 among 80 adult patients infected with SARS-CoV-2 and diagnosed with COVID-19 in Imam Abdulrahman Alfaisal hospital’s inpatient department in Riyadh, Saudi Arabia. The criteria for diagnosing COVID-19 were based on confirming a positive RT-PCR test for the SARS-CoV-2 after taking a throat or nose swab sample from each patient. Patients who started on antiviral medication were not eligible for this study.

Within 24 hours of confirming the diagnosis and hospital admission, patients have been divided into Group-A and Group-B depends on the disease severity. Blood samples were drawn from each patient to measure the serum levels of copper, iron “in the form of ferritin,” selenium, and zinc. All patients included were admitted during the first days of the symptomatic period and required hospitalization; however, the beginning of the incubation period and infection period of SARS-CoV-2 was unknown. Group-A includes non-severe cases of COVID-19, which is defined as patient’s no need for oxygen therapy and not diagnosed with pneumonia. Group-B includes severe cases, which is defined as patients had one of the following criteria: respiratory distress with respiratory frequency ≥30/min, pulse oximeter oxygen saturation ≤93% at rest or oxygenation index (artery partial pressure of oxygen/inspired oxygen fraction, PaO2/FiO2) ≤300 mm Hg. The diagnosis of COVID-19 severity was made according to the diagnostic and treatment guidelines for SARS-CoV-2 issued by the Chinese National Health Committee (http://en.nhc.gov.cn/2020-03/29/c_78469.htm) [14].

## 3. Data collection

Blood was drawn from 80 hospitalized patients diagnosed with COVID-19. For micronutrients (copper, iron “in the form of ferritin,” selenium, and zinc) blood serum levels, blood samples were collected using blood collecting tubes with no additives or anticoagulants. The samples were sent to delta company for medical laboratories in Riyadh, which is accredited by the College of American Pathologists (CAP) and certified by the International Organization for Standardization (ISO 9001:2015) with the collaboration of The Doctors Laboratory (TDL) in the United Kingdom. The blood plasma concentrations of the mentioned micronutrients have been measured in patients from both study groups, Group-A and Group-B. In addition to the mentioned trace elements, other tests were done in the hospital, including white blood cell count (WBCs), red blood cell count (RBCs), sodium (Na), potassium (K), chloride (Cl), and calcium (Ca) blood concentrations. Non-personal information includes nationality, gender, weight, height, and age were collected from patients’ files. Symptoms, pulse oximeter oxygen saturation, respiratory rate, oxygen therapy requirement, medical history, and medication history of each included patients were reported by specialized nurse staff.

## 4. Endpoints

This study’s primary endpoint was to determine the correlation between disease severity and trace elements’ serum levels among COVID-19 patients.

## 5. Statistical analysis

The data saved, organized, and graphed by using Microsoft excel 2010 program. The association between zinc, iron, copper, and selenium blood plasma levels and the severity of symptoms of patients infected with SARS-CoV-2 have been determined by using the chi-square test, student t-test, and Mann–Whitney U test. The level of significance will be considered at P < 0.05.

## 6. Results

Eighty patients diagnosed with COVID-19 had been enrolled in this study after their acceptance to participate, and blood samples were taken from them. Forty-five (45) of them were non-severe cases, and 35 were severe cases of COVID-19. The mean age was 51.54 years old, and the mean body mass index (BMI) was 30 kg/m^2^. The percentage of Saudi patients was 54%, and 64.9% of the patients were male. The percentage of patients with non-severe cases of COVID-19 was 56.25%, and 47% of them had a fever. However, patients with severe cases were 43.75%, and 85% had a fever. There was a significant difference between non-severe and severe cases regarding fever (P-value < 0.0003). Shortness of breath (SOB) symptom was observed in 95% among severe cases and 35.3% among non-severe cases, and the difference between them was significant (P-value < 0.0001). Seventy percent (70%) of patients with severe COVID-19 conditions were older than 50 years, and 64.7% of patients with non-severe conditions were younger than 50 years (P-value < 0.0003). The percentages of diabetic and hypertensive patients in this study were 45.9% and 64.9%, respectively, and 66.6% of hypertensive patients were severe cases, where 33.3% of them were non-severe cases (P-value < 0.002). Other patients’ characteristics are shown in Table 1.

**Table 1.** Baseline characteristics of patients

Variables	All COVID-19 cases (N = 80)	Non-severe cases (N = 45)	Severe cases (N = 35)	P-value
Mean age (in years)	51.54	43.7	58.2	0.0001
Older than 50 years (%)	54	30	70	0.0003
Mean body mass index (kg/m^2^)	30	29.2	31.3	0.19
Saudi nationality (%)	54	58.8	50	0.57
Fever (%)	67.6	47	85	0.0003
Shortness of breath (SOB) (%)	67.6	35.3	95	0.0001
Respiratory rate (breath per minute)	25	21.5	28	0.0001
Diabetes (%)	45.9	41.2	50	0.57
Hypertension (%)	64.9	47	80	0.002
ACEI/ARBs use (%)	32.4	29.4	35	0.6
Antibacterial use (%)	64.9	64.7	65	0.9
Anti-platelet or anti-coagulant use (%)	64.9	52.9	75	0.055

Regarding lab tests including WBCs, RBCs, Na, and K, there were no significant differences between non-severe and severe COVID-19 patients with mean values showed 9010 cells/mm3 (9941 vs 8079 cells/mm3, P-value = 0.17), 4.66 million/mm3 (4.55 vs 4.78 million/mm3, P-value = 0.2), 137.25 mmol/l (138 vs 136.58 mmol/l, P-value = 0.16) and 4.3 mmol/l (4.4 vs 4.2 mmol/l, P-value = 0.15) respectively. The exception was for calcium and chloride plasma levels, since it was revealed that severe cases had hypocalcemia and lower concentrations of chloride in blood plasma. The differences of calcium and chloride non-severe and severe COVID-19 patients with mean values showed 2.04 mmol/l (2.11 vs 1.99 mmol/l, P-value < 0.01) and 100.29 mmol/l (103.3 vs 97.7 mmol/l, P-value < 0.0014), respectively (see Table 2).

**Table 2.** Mean levels of some lab tests among patients diagnosed with severe and non-severe symptoms of COVID-19

Lab tests -mean values-	All COVID-19 cases (N = 80)	Non-severe cases (N = 45)	Severe cases (N = 35)	P-value
WBCs (cells/mm^[3]^)	9010	9941	8079	0.17
RBCs (million/mm^[3]^)	4.66	4.55	4.78	0.2
Na (mmol/L)	137.25	138	136.58	0.16
K (mmol/L)	4.3	4.4	4.2	0.15
Ca (mmol/L)	2.04	2.11	1.99	0.01
Cl (mmol/L)	100.29	103.3	97.7	0.0014

**Figure 1.**
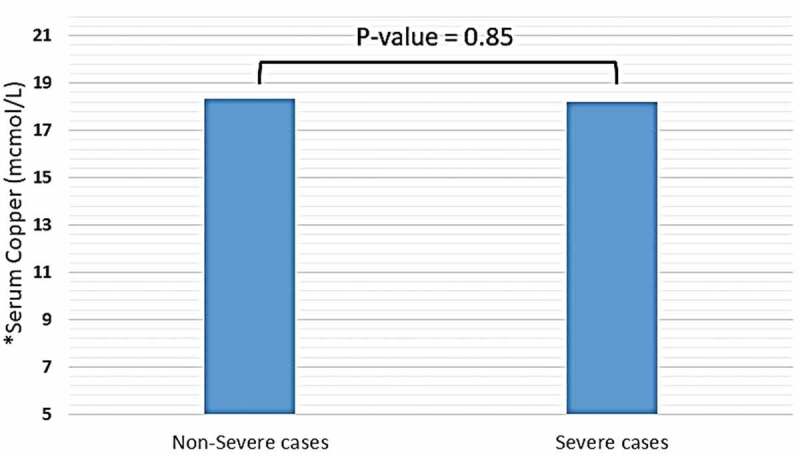
Mean serum level of copper among patients diagnosed with severe and non-severe symptoms of COVID-19. * Normal range of copper in blood serum: 10–22 mcmol/L

**Figure 2.**
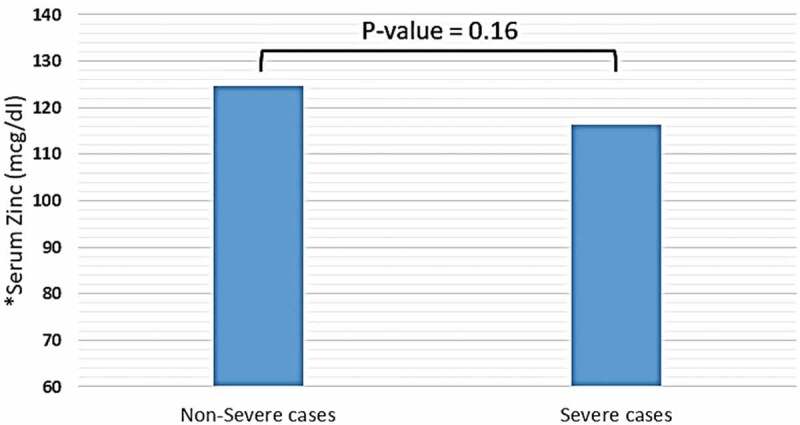
Mean serum level of zinc among patients diagnosed with severe and non-severe symptoms of COVID-19. * Normal range of zinc in blood serum: 66–110 mcg/dl

**Figure 3.**
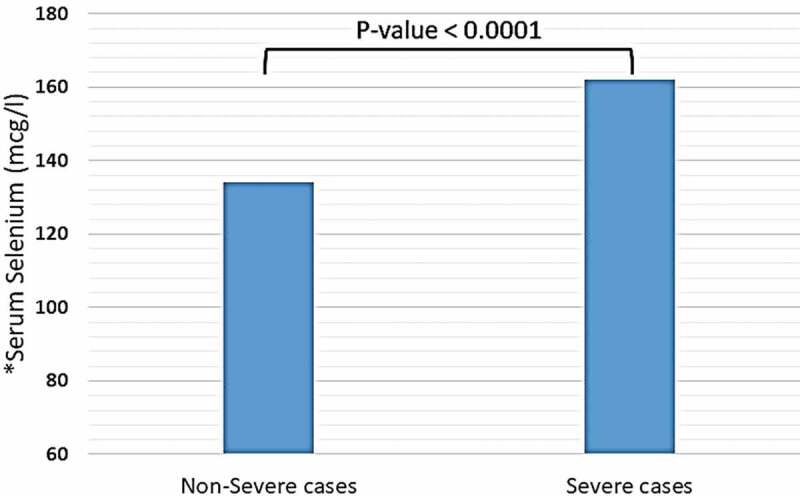
Mean serum level of selenium among patients diagnosed with severe and non-severe symptoms of COVID-19. * Normal range of selenium in blood serum: 70–150 mcg/L

**Figure 4.**
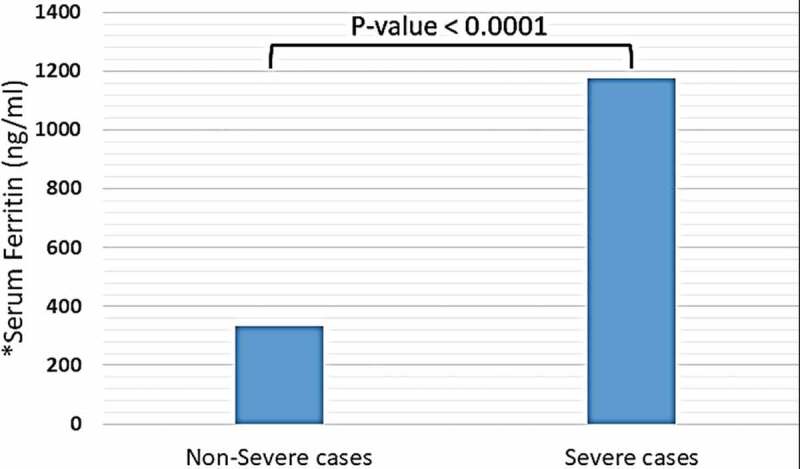
Mean serum level of ferritin among patients diagnosed with severe and non-severe symptoms of COVID-19. * Normal range of ferritin in blood serum: 12–300 ng/ml

Trace elements, including copper, iron (in the form of ferritin), selenium, and zinc were detected in distinct blood plasma levels among non-severe and severe COVID-19 patients. In both two study groups, the mean copper serum level was within the normal range (18.2 mcmol/l), and the difference between non-severe and severe cases regarding mean copper serum levels of each group was not significant (18.35 vs. 18.2 mcmol/l, P-value = 0.85) (see Figure 1). Unlike copper; mean zinc serum level among both study groups was slightly elevated-based Gibson RS et al. [15] (121.78 mcg/dl), and the differences between non-severe and severe cases were not significant (124.57 vs. 116.37 mcg/dl, P-value = 0.16) (see Figure 2). Nevertheless, the mean selenium in both study groups was within the normal range (138 mcg/l). However, there was a significant difference in selenium mean serum level between non-severe and severe cases, which showed significant elevation selenium level among severe cases of COVID-19 patients (134 vs. 162 mcg/l, P-value < 0.0001) (see Figure 3). Mean ferritin serum level among COVID-19 patients in this study was elevated (776.53 ng/ml), and there was a vast difference between non-severe and severe cases regarding ferritin mean levels (331.24 vs. 1174.95 ng/ml, P-value < 0.0001) (see Figure 4 and Table 3).

**Table 3.** Mean serum levels of copper, ferritin, selenium, and zinc among patients diagnosed with severe and non-severe symptoms of COVID-19

Trace elements -mean values-	Normal Values	All COVID-19 cases (N = 80)	Non-severe cases (N = 45)	Severe cases (N = 35)	P-value
Copper (mcmol/L)	10–22	18.2	18.35	18.2	0.85
Iron -in the form of ferritin- (ng/ml)	12–300	776.53	331.24	1174.95	< 0.0001
Selenium (mcg/L)	70–150	138	134	162	< 0.0001
Zinc (mcg/dl)	66–110	121.78	124.57	116.37	0.16

## 7. Discussion

Trace elements, including zinc, copper, iron, selenium, chromium, boron, iodine, manganese, molybdenum, and vanadium, are components of human cells found in blood serum with low quantity. Even though these minerals are substantial for the cell’s normal function, most trace elements act as cofactors for several enzymes. Trace elements are divided into essential and non-essential trace elements for human cells. Copper, iron, selenium, and zinc are classified as essential trace elements in human cells. Any excess or deficiency of these four elements leads to mild to severe symptoms or even death [16].

During bacterial, viral, or fungal infections, different cell types damage and disturb mRNA expression of many genes due to the systemic inflammation and increase the release of cytokines that lead to cytokine storm syndrome [17]. Many previous studies did not test the levels of trace elements in blood serum during viral infections and how they could correlate with adverse outcomes [18]. Some studies revealed that copper and ferritin serum levels were increased during several viral infections caused by Human Immunodeficiency Virus-1 (HIV-1), Hepatitis C Virus (HCV), and human influenza virus. Iron overload during viral infection may facilitate and promote viral replication that provokes more adverse effects and could lead to poor prognosis and even death [19]. Other studies found that serum level of copper elevated in the presence of some pathogens, including mycobacterium tuberculosis, streptococcus pneumonia, and Cryptococcus neoformans. These microorganisms utilize copper to activate many enzymes responsible for pathogen reproduction and to replace iron clusters in the host to deactivate other types of enzymes [20].

In this study, researchers measured the serum level of four essential trace elements including copper, iron, selenium, and zinc among patients diagnosed with COVID-19 in Riyadh, which revealed that patients complain of low oxygen saturation and rapid respiratory rate symptoms were frequently and significantly increased along with iron and selenium serum levels increased. However, zinc serum was elevated among COVID-19 patients. There was no significant difference between both study groups. These results indicate possible changes in human cells during viral infection with SARS-CoV-2. Some of these changes could be due to mutations of genes responsible for the expression of transporters for iron, selenium, or zinc, altering these trace elements’ metabolism, affecting the oxygen transport system, and increasing oxidative stress alters immune system functions.

**Figure 5.**
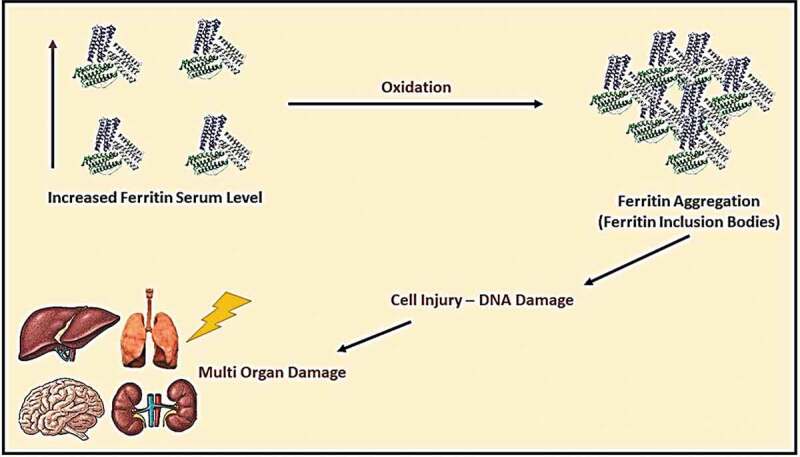
Ferritin metabolism and pathway in causing multi-organ inflammation and damage

**Figure 6.**
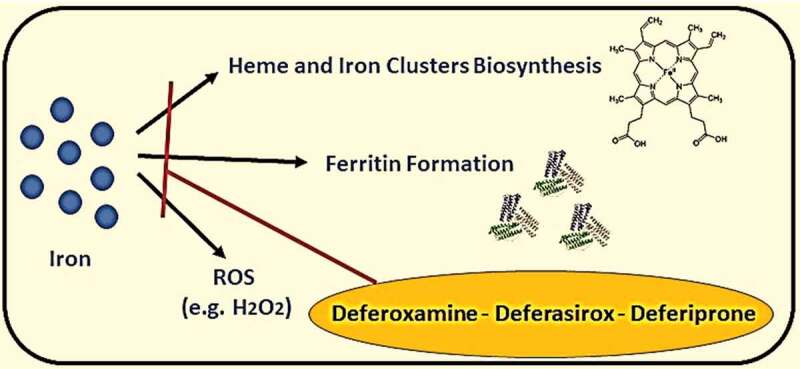
Products generated by free iron elements and drugs (deferoxamine, deferasirox or deferiprone) inhibiting its pathways. *ROS: Reactive Oxygen Species

**Figure 7.**
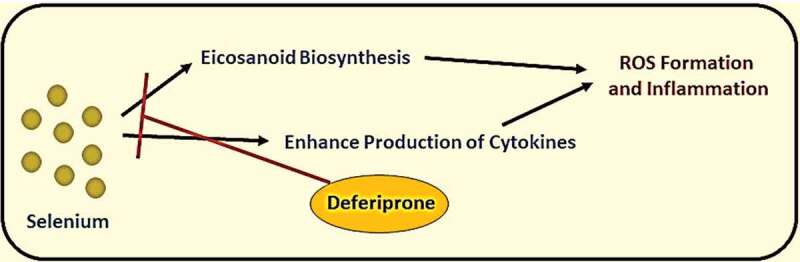
Products generated by free selenium elements and drug (deferiprone) inhibiting its pathways

It was known that iron, selenium, and zinc are essential micronutrients for human health; however, these elements’ excess levels could cause adverse reactions. The elevation of iron serum above the normal level may lead to ferritin aggregation, causing shortness of breath, fatigue, general pain, and systemic inflammation (see Figure 5) [21]. On the other hand, selenium’s elevation may lead to malfunction of different cells due to ER stress and increase biosynthesis of pro-inflammatory prostaglandins. Nevertheless, an elevated level of serum zinc could cause DNA damage and mutations, and malfunction of immune cells. The treatment of elevated trace elements by giving polyvalent chelators (e.g., Deferiprone) could reduce reactive oxygen species (ROS) generation because of excess iron and selenium in blood serum during hyperinflammatory state caused by a severe viral infection (see Figure 6 and Figure 7) [22,23].

### 7.1. Limitations

This study’s small sample size was considered one of its limitations, and the results are at an 80% confidence level. Besides, no records were found regarding nutritional status or gastrointestinal symptoms for the included patients.

## 8. Conclusion and recommendations

### 8.1. Conclusion

Iron and zinc serum levels were elevated among patients infected by SARS-CoV-2 and diagnosed with COVID-19. There was a significant elevation of selenium and iron serum levels among severe cases compared to non-severe cases of COVID-19. High levels of iron and selenium could be correlated with the disease severity during infection with SARS-CoV-2.

### 8.2. Recommendation

Since this study revealed significant deviations of some trace elements in blood serum during the first days of patients’ admission, the researchers suggest to expect variations of trace elements during other stages of SARS-CoV-2 infection and recommend further studies to confirm the findings reached in the current study.
